# Phylogenetic position of the langur genera *Semnopithecus *and *Trachypithecus *among Asian colobines, and genus affiliations of their species groups

**DOI:** 10.1186/1471-2148-8-58

**Published:** 2008-02-25

**Authors:** Martin Osterholz, Lutz Walter, Christian Roos

**Affiliations:** 1 Department of Primate Genetics, German Primate Center, Kellnerweg 4, 37077 Goettingen, Germany; 2 Gene Bank of Primates, German Primate Center, Kellnerweg 4, 37077 Goettingen, Germany

## Abstract

**Background:**

The evolutionary history of the Asian colobines is less understood. Although monophyly of the odd-nosed monkeys was recently confirmed, the relationships among the langur genera *Presbytis*, *Semnopithecus *and *Trachypithecus *and their position among Asian colobines remained unclear. Moreover, in *Trachypithecus *various species groups are recognized, but their affiliations are still disputed. To address these issues, mitochondrial and Y chromosomal sequence data were phylogenetically related and combined with presence/absence analyses of retroposon integrations.

**Results:**

The analysed 5 kb fragment of the mitochondrial genome allows no resolution of the phylogenetic relationships among langur genera, but five retroposon integrations were detected which link *Trachypithecus *and *Semnopithecus*. According to Y chromosomal data and a 573 bp fragment of the mitochondrial cytochrome b gene, a common origin of the species groups *T*. [*cristatus*], *T*. [*obscurus*] and *T*. [*francoisi*] and their reciprocal monophyly is supported, which is also underpinned by an orthologous retroposon insertion. *T*. [*vetulus*] clusters within *Semnopithecus*, which is confirmed by two retroposon integrations. Moreover, this species group is paraphyletic, with *T. vetulus *forming a clade with the Sri Lankan, and *T. johnii *with the South Indian form of *S. entellus*. Incongruence between gene trees was detected for *T*. [*pileatus*], in that Y chromosomal data link it with *T*. [*cristatus*], *T*. [*obscurus*] and *T*. [*francoisi*], whereas mitochondrial data affiliates it with the *Semnopithecus *clade.

**Conclusion:**

Neither relationships among the three langur genera nor their position within Asian colobines can be settled with 5 kb mitochondrial sequence data, but retroposon integrations confirm at least a common origin of *Semnopithecus *and *Trachypithecus*. According to Y chromosomal and 573 bp mitochondrial sequence data, *T*. [*cristatus*], *T*. [*obscurus*] and *T*. [*francoisi*] represent true members of the genus *Trachypithecus*, whereas *T*. [*vetulus*] clusters within *Semnopithecus*. Due to paraphyly of *T*. [*vetulus*] and polyphyly of *Semnopithecus*, a split of the genus into three species groups (*S. entellus *- North India, *S. entellus *- South India + *T. johnii*, *S. entellus *- Sri Lanka + *T. vetulus*) seems to be appropriate. T. [*pileatus*] posses an intermediate position between both genera, indicating that the species group might be the result of ancestral hybridization.

## Background

The Old World monkeys are traditionally divided into the two subfamilies Cercopithecinae and Colobinae, which differ from each other by numerous morphological, behavioural and ecological characteristics [1-4]. While detailed information on the evolutionary history of cercopithecines (baboons, mangabeys, macaques and guenons) is at hand, knowledge on colobines is still scarce. Although some molecular studies [5-13] exist, they mainly focus on relationships within genera or species groups and not on the general phylogeny of the subfamily. Recently, a first mitochondrial phylogeny of colobine genera was established [14], which confirmed some previous assumptions, but also led to confusions, calling for further research to definitively elucidate their evolutionary history.

Based on distribution and morphology, the colobines are traditionally divided into an African and an Asian clade [3,4], while Asian colobines are more diverse than their African relatives. Hence, the Asian forms are further split into the odd-nosed monkey (*Pygathrix, Rhinopithecus, Nasalis, Simias*) and langur (*Presbytis, Trachypithecus, Semnopithecus*) groups, which are both believed to be monophyletic. Accordingly, langurs were originally combined in the single genus *Presbytis *[15-17] or *Semnopithecus *[18], but based on neonatal colouration and cranial morphology, they were split into the three genera *Semnopithecus, Trachypithecus *and *Presbytis *[19], and a fourth genus (*Kasi*) was added [20]. Alternatively, *Semnopithecus *was separated from *Presbytis*, with *Trachypithecus *forming a subgenus of the former [21-23], but recent classifications use a subdivision of langurs into the three genera *Presbytis, Trachypithecus *and *Semnopithecus *[3,4,24-26].

**Figure 1:**
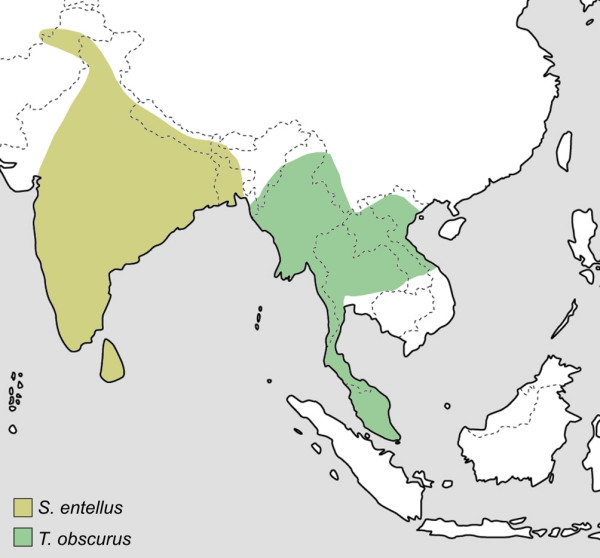
**Distribution of the genus *Semnopithecus *and *Trachypithecus *[*obscurus*]**. Genus affiliations and species groups after Groves [4].

**Figure 2:**
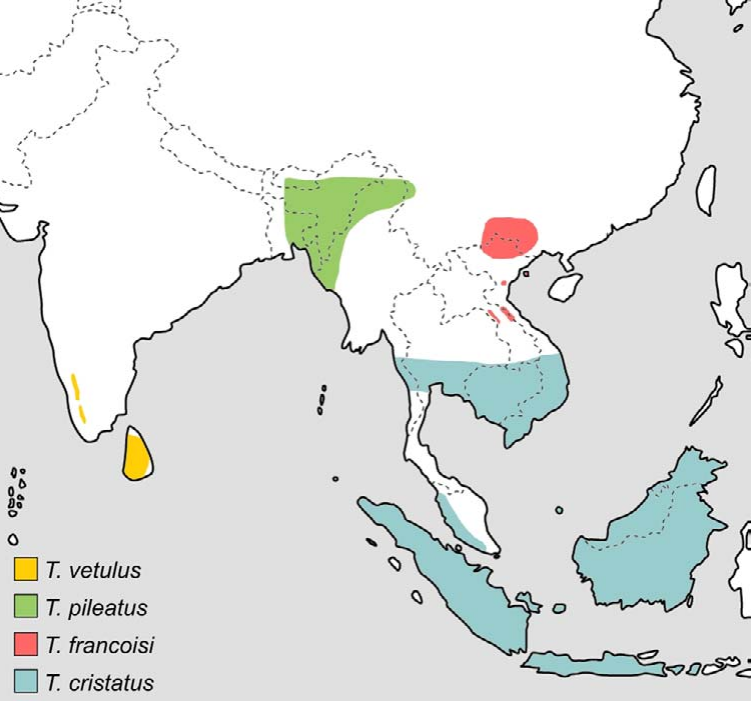
**Distribution of the *Trachypithecus *species groups *T*. [*vetulus*], *T*. [*pileatus*], *T*. [*francoisi*] and *T*. [*cristatus*]**. Genus affiliations and species groups after Groves [4].

Within the different langur genera, several species are recognized, which are lumped into species groups due to similar fur colouration, behaviour, ecology or distribution. With five species groups (*T*. [*obscurus*], *T*. [*francoisi*], *T*. [*cristatus*], *T*. [*pileatus*] and *T*. [*vetulus*]) [4], the genus *Trachypithecus *is the most diverse of all langurs and posses also the widest distribution, ranging from South India and Sri Lanka through mainland Southeast Asia to the Sundaland (Fig. 1, Fig. 2). Although all of them are morphologically similar, *T*. [*vetulus*] was sometimes separated in its own genus *Kasi *[20], and recent mitochondrial sequence data indicate a closer affiliation of *T*. [*vetulus*] and *T*. [*pileatus*] to *Semnopithecus *than to *Trachypithecus *[7,9]. Accordingly, the two *T*. [*vetulus*] members were recognized as species of *Semnopithecus *[26]. In contrast to *Trachypithecus*, the genus *Semnopithecus *is restricted to the Indian subcontinent (Fig. 1) and traditionally regarded as monotypic with the only species *S. entellus *[3,27], although recently several subspecies were elevated to species status [4]. The third langur genus, *Presbytis*, includes several species, which occur solely in the Sundaland, but are not lumped into distinct species groups [4].

The phylogenetic relationships among the different Asian colobine genera are disputed. Although a common origin of the odd-nosed monkeys was recently confirmed [14], evidence for monophyly of its putative sister clade, the langur group, is still lacking. Moreover, available data depict *Trachypithecus *and *Presbytis *as sister taxa to the exclusion of *Semnopithecus *[14], which contradicts with traditional classifications, in which *Trachypithecus *and *Semnopithecus *are believed to form a clade to the exclusion of *Presbytis *[4,21-23]. These findings raise the question of what positions the langur genera occupy among Asian colobines and whether the langurs form a monophyletic clade in general. Moreover, the affiliations of different *Trachypithecus *species groups, especially *T*. [*vetulus*] and *T*. [*pileatus*], are disputed, and hence, led to different classifications. Currently, only few genetic data are available [7,9,28], so that further information from other markers is required to definitively establish their relationships.

To address all these issues, mitochondrial and Y chromosomal sequence data were phylogenetically related and combined with presence/absence analysis of retroposon integrations. This approach was used to simultaneously analyse paternal-, maternal- and biparental-inherited markers, which allow the detection of incongruence between different gene trees indicating possible hybridization or introgression events between different lineages [29-31]. To determine the phylogenetic position of the langur genera among Asian colobines, a 5 kb fragment of the mitochondrial genome was sequenced from eight colobine genera, and combined with presence/absence analysis of retroposon integrations. Retroposon insertion events are nearly homoplasy-free and precise excision of elements is highly unlikely [32,33]. Accordingly, retroposon insertions are powerful informative markers, which were already successfully applied to elucidate phylogenetic relationships in various primate lineages [34-38]. To study relationships among different langur species groups and their genus affiliations, a 573 bp fragment of the mitochondrial cytochrome b gene and a 777 bp portion of the SRY (sex-determining region, Y chromosome) gene was sequenced from at least one representative per species group, and complemented with retroposon analysis.

## Results and Discussion

### Genus level phylogeny

To elucidate the phylogenetic relationships among the different langur genera and their position among Asian colobines, mitochondrial sequence studies were combined with presence/absence analysis of retroposon insertions.

The herein analysed 5 kb fragment of the mitochondrial genome was assembled from sequences derived from 1–2 kb long and partly overlapping PCR products, whereby no inconsistencies in overlapping sequence fragments were detected. As template material, mainly DNA extracted from feces was used, which minimizes the amplification of nuclear pseudogenes [39], and comparisons of the data with sequences already deposited at GenBank revealed only intra-species or -generic variation, indicating that no nuclear pseudogenes were amplified.

**Figure 3:**
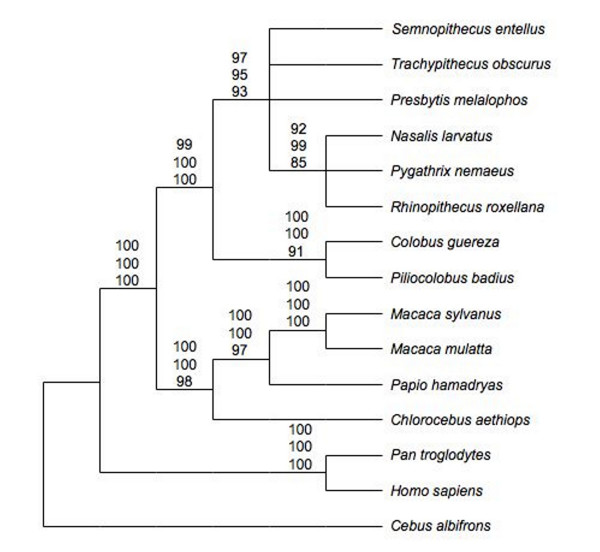
**Phylogenetic relationships among colobines and related genera based on mitochondrial data**. Numbers on nodes indicate support values >80% (first: ML, second: NJ, third: MP).

To determine the phylogenetic relationships among analysed genera, tree reconstructions were conducted with different algorithms, which all led to the same tree topology (Fig. 3). Moreover, most relationships are significantly supported and congruent with previous classifications [1-4], indicating the reliability of the data set. In detail, the reconstructions confirm relationships among cercopithecine genera (*Macaca, Papio, Chlorocebus*), and reciprocal monophyly of cercopithecines and colobines, as well as of African and Asian colobines. Within the Asian clade, relationships are not resolved, although at least a common origin of the odd-nosed monkeys is depicted. Neither the assumed monophyly of the langur genera, nor the expected close affinity of *Trachypithecus *and *Semnopithecus *or the recently indicated sister grouping of *Trachypithecus *and *Presbytis *[14] can be verified with significance. Moreover, all alternative tree topologies, in which the three langur genera were regarded as monophyletic, or variously recognized as sister clades to each other, to the odd-nosed monkey clade, or even as basal among Asian colobines, were not rejected (P = 0.097 – 0.776). Accordingly, an unresolved polytomy among the three langur genera and the odd-nosed monkey clade may best reflect the relationships among Asian colobines based on the mitochondrial data.

**Figure 4:**
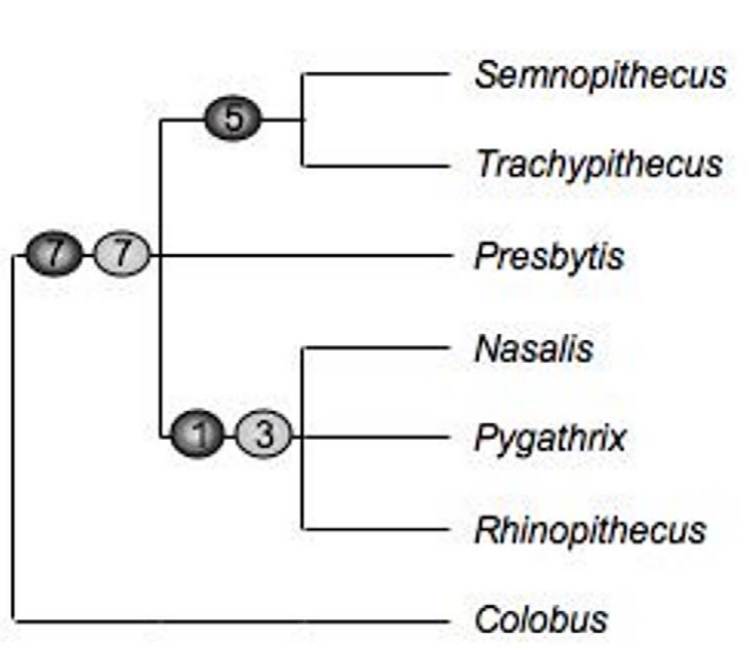
**Phylogenetic relationships among Asian colobine genera based on retroposon integrations**. Dark dots represent new generated data, whereas light dots refer to already published data [37]. Numbers in dots indicate single integration events.

Several retroposon insertions were detected, which provide insights into the branching pattern of colobines (Fig. 4). Together with seven already published integrations [38], now 14 loci confirm monophyly of the Asian colobines to the exclusion of the African genus *Colobus*. Among Asian colobines, one insertion indicates a common origin of the odd-nosed monkeys, which is verified by three further loci [38]. Most prominent are the five retroposon integrations, which confirm a sister grouping of *Trachypithecus *and *Semnopithecus*. No loci were detected, which link *Presbytis *either with the other two langur genera or the odd-nosed monkeys.

Although in general the mitochondrial data are suitable to elucidate relationships among the different genera, as indicated by the correct and significantly supported branching patterns among all other studied genera, the relationships among the langurs are unresolved, which is concordant with previous results [14]. In contrast, the presence/absence analysis of retroposon integrations provides evidence for a monophyletic odd-nosed monkey clade and a common origin of *Trachypithecus *and *Semnopithecus*, which is in agreement with morphological hypotheses [1,3,4,21-23]. Regardless which markers were used, the phylogenetic position of *Presbytis *among Asian colobines and accordingly the unity of the langurs remains unclear and needs further investigations.

### Species group phylogeny

In order to settle affiliations among the different *Trachypithecus *species groups and their members, mitochondrial and Y chromosomal sequence data were combined with information on retroposon integrations.

**Figure 5:**
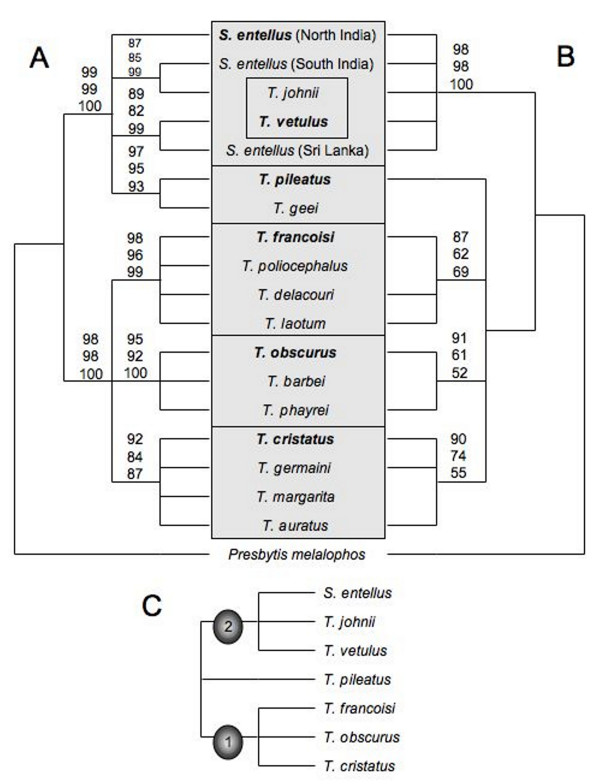
**Phylogenetic relationships among *Semnopithecus *and *Trachypithecus *species groups based on a) mitochondrial data, b) Y chromosomal data, and c) retroposon integrations**. Numbers on nodes indicate support values (first: ML, second: NJ, third: MP), and boxed species belong to a species group, with species in bold giving the name of the group.

The mitochondrial phylogeny was established on the basis of 573 bp long cytochrome b gene sequences, generated from most species recognized in the genus and its sister genus *Semnopithecus*. In all tree reconstructions, identical relationships were obtained, with most branches being significantly supported (Fig. 5a). Accordingly, the different species groups are divided into two major groups, with one including solely groups of the genus *Trachypithecus*, whereas the second one includes representatives of *Trachypithecus *and *Semnopithecus*. In the mixed clade, *T*. [*vetulus*] and *T*. [*pileatus*] members are lumped together with *S. entellus*. Whereby *T*. [*pileatus*] is monophyletic, the members of *T*. [*vetulus*] are paraphyletic, with *T. vetulus *forming a sister clade to *S. entellus *from Sri Lanka, and *T. johnii *with *S. entellus *from South India. Furthermore, a fourth lineage in the mixed clade was detected, which is represented by *S. entellus *from North India. In contrast, the three species groups (*T*. [*obscurus*], *T*. [*francoisi*], *T*. [*cristatus*]) in the clade comprising solely *Trachypithecus *groups are all monophyletic. Alternative relationships, in which *T*. [*vetulus*] is recognized as monophyletic, either *T*. [*vetulus*] or *T*. [*pileatus*] belongs to *Trachypithecus *or even both are members of *Trachypithecus*, were tested, but all of them were rejected (P < 0.05).

With some exceptions, the Y chromosomal data provide a similar picture (Fig. 5b), but due to the low number of polymorphic sites, support values are in general not as high as in the mitochondrial tree. According to the reconstructions, the species groups are divided into two major clades, with one comprising *T*. [*obscurus*], *T*. [*cristatus*], *T*. [*francoisi*] and *T*. [*pileatus*], and the other, *T*. [*vetulus*] and *Semnopithecus*. Relationships among the latter are not resolved. All alternative tree topologies, in which either *T*. [*vetulus*] belongs to *Trachypithecus *or *T. pileatus *groups with *Semnopithecus*, were rejected (P < 0.05).

Retroposon insertions further deepened our knowledge on the species group relationships. Altogether, three informative integrations were analysed (Fig. 5c), with one occurring in *T*. [*obscurus*], *T*. [*cristatus*] and *T*. [*francoisi*], and the other two in *T*. [*vetulus*] and *Semnopithecus*. Interestingly, all three integrations are absent in *T. pileatus*.

With the exception of the varying position of *T*. [*pileatus*], the affiliations of the remaining species groups are congruent among different gene trees. Accordingly, all analysed markers relate *T*. [*vetulus*] with *Semnopithecus*, indicating that this species group is a real member of the genus *Semnopithecus *and not of *Trachypithecus *as assumed by morphological similarities [4]. These similarities may be the results of adaptations to similar ecological conditions (*Semnopithecus *is semi-terrestrial and lives in deciduous forest, whereas *Trachypithecus *including *T*. [*vetulus*] is arboreal and occurs in wet evergreen forests). Although the Y chromosomal data allow no resolution within the *Semnopithecus *– *T*. [*vetulus*] clade, the mitochondrial data indicate paraphyly of the two *T*. [*vetulus*] species, with *T. vetulus *clustering with *S. entellus *from Sri Lanka and *T. johnii *with *S. entellus *from South India, which is concordant with their geographical distribution. These findings indicate paraphyly of *S. entellus*, whereby North Indian representatives form a further distinct lineage. Accordingly, the langurs of the Indian subcontinent should be split into three species groups, with one occurring solely on Sri Lanka, one in Southern India and a third one in Northern India, whereas the Gondavari river seems to be barrier between the latter two.

Monophyly of each of *T*. [*obscurus*], *T*. [*cristatus*] and *T*. [*francoisi*] and their close affiliation is depicted in all gene trees, so that all of them can be regarded as true members of *Trachypithecus*. These findings confirm previous molecular studies [8-11,13] and are in general agreement with recent classifications [4,26].

The only discrepancies between different gene trees were obtained for *T*. [*pileatus*]. Whereas the mitochondrial data link the species group with *Semnopithecus *and *T*. [*vetulus*], the Y chromosomal data affiliates it with *T*. [*obscurus*], *T*. [*cristatus*] and *T*. [*francoisi*]. These findings might be explained by incomplete lineage sorting of ancestral mitochondrial or Y chromosomal haplotypes. Accordingly, the ancestor of *Trachypithecus, Semnopithecus *and *T*. [*pileatus*] carried multiple DNA lineages with one lineage being randomly fixed in two taxa, but not in the third. Alternatively, the varying position of *T*. [*pileatus*] in different gene trees might be explained by past hybridization between *Semnopithecus *and *Trachypithecus*. As depicted by the three retroposon insertions, this putative hybridization event would have occurred between ancestral forms of *Semnopithecus *and *Trachypithecus*, before both genera diverged into distinct species groups. The hybridization hypothesis is also supported by some intermediate morphological characteristics [4] and the distribution of *T*. [*pileatus*], which is sandwiched between those of *Semnopithecus *and other *Trachypithecus *species groups (Fig. 1, Fig. 2).

## Conclusion

The present study provides detailed insights into the evolutionary history of Asian colobines and underpins the tremendous power of retroposon integrations as cladistic markers. Although mitochondrial data proved to be useful to elucidate and confirm several relationships among studied taxa, the data set failed to resolve the affiliations among the langur genera and to settle their position among Asian colobines. In contrast, retroposon insertions provided clear evidence for a sister grouping of *Semnopithecus *and *Trachypithecus*, but no integrations were detected, which link *Presbytis *either with the other two langur genera or with the odd-nosed monkeys, so that further research is required to solve this issue. Moreover, to definitively explain the evolutionary history of colobines, further molecular markers should be analysed, especially regarding possible discrepancies among gene trees due to hybridization or introgression, as such events are important speciation mechanisms in primates [31], and as it was possibly detected in the present study in the case of *T*. [*pileatus*].

Although further investigations are necessary to fully understand the evolution of colobines, the present study provides sufficient data to revise the current classification of the genera *Trachypithecus *and *Semnopithecus *(Table 1). Accordingly, within *Trachypithecus*, three reciprocal monophyletic species groups (*T*. [*obscurus*], *T*. [*cristatus*] and *T*. [*francoisi*]) should be recognized, whereas *T*. [*vetulus*] should be included in *Semnopithecus*. Moreover, due to their paraphyletic origin, *T. vetulus *and *T. johnii *should not be lumped into a single species group, and *S. entellus *should not be regarded as monotypic. A classification into three species groups with one occurring in Northern India, one in Southern India and one on Sri Lanka may best reflect the evolutionary relationships among the langurs of the Indian subcontinent. However, this arrangement is tentative, because further research is required to confirm their distinctiveness not only on mitochondrial, but also on nuclear DNA level. *T*. [*pileatus*] might be the result of an ancient hybridization event between *Semnopithecus *and *Trachypithecus*, and hence, its classification is difficult. While a separation in its own distinct genus may be appropriate, we provisionally accept its traditional recognition as member of *Trachypithecus*.

**Table 1:** Proposed classification of *Semnopithecus *and *Trachypithecus *species based on the herein presented data.

Species group	*Semnopithecus*	*Trachypithecus*
S. entellus* (North India)	S. entellus*	
S. entellus* (South India)	S. entellus*	
	*S. johnii*	
S. entellus* (Sri Lanka)	S. entellus*	
	*S. vetulus*	

T. pileatus**	*T. pileatus*	
	*T. geei*	

*T. francoisi*		*T. francoisi*
		*T. poliocephalus*
		*T. delacouri*
		*T. laotum*
*T. obscurus*		*T. obscurus*
		*T. barbei*
		*T. phayrei*
*T. cristatus*		*T. cristatus*
		*T. germaini*
		*T. margarita*
		*T. auratus*

* species/subspecies designation has to be assessed.

** provisionally classified as member of *Trachypithecus*.

## Methods

### Sample collection, DNA extraction and preventing contaminations

The species analysed in this study are presented in Table 2. All study specimens were identified by fur colouration and other external characteristics. Hanuman langur (*S. entellus*) samples were collected only from founder animals, of which the area of capture was at least roughly known. Total genomic DNA was extracted from blood, tissue or feces using the DNeasy or Stool Mini Kits from Qiagen. When hair follicle cells were used, 1–3 hairs were directly implemented into the PCR reaction after they were washed with sterile water and 95% ethanol. To prevent contaminations, sample collection and laboratory procedures followed described standard protocols [40-43]. In detail, all fecal and hair samples were collected with gloves and stored in sterile tubes or plastic bags before further processing. DNA extraction, PCR, gel extraction and sequencing was performed in separate laboratories and repeated after several months, while always only one individual per species was tested. Moreover, from most specimens two different sample types were available, which both were used as template material. Sequences from independent analyses were identical. Finally, all PCR reactions were performed with negative (distilled water) controls.

**Table 2:** Species analysed, their origin, material type and GenBank accession numbers.

Species	Origin	Material type	mtDNA (5 kb)	mtDNA (573 bp)	SRY (777 bp)
*Cebus albifrons*	GenBank	sequence	NC_002763	-	-
*Homo sapiens*	GenBank	sequence	X93334	-	-
*Pan troglodytes*	GenBank	sequence	NC_001643	-	-
*Chlorocebus aethiops*	GenBank	sequence	NC_007009	-	-
*Papio hamadryas*	GenBank	sequence	NC_001992	-	-
*Macaca mulatta*	GenBank	sequence	NC_005943	-	-
*M. sylvanus*	GenBank	sequence	NC_002764	-	-
*Colobus guereza*	Cologne Zoo	tissue, feces	EU004483	-	-
*Piliocolobus badius*	MPI Leipzig	tissue, feces	EU004482	-	-
*Pygathrix *nemaeus	Cologne Zoo	tissue, feces	EU004481	-	-
*Rhinopithecus avunculus*	EPRC	tissue	EU004480	-	-
*Nasalis larvatus*	Wilhelma Stuttgart	blood	EU004476	-	-
*Presbytis melalophos*	Howletts Zoo	tissue, feces	EU004479	part of 5 kb	EU004456
*Semnopithecus entellus *(North India)	Dresden Zoo	blood, feces	EU004478	part of 5 kb	EU004457
*S. entellus *(South India)	Hannover Zoo	blood, feces	-	EU004471	EU004458
*S. entellus *(Sri Lanka)	Krefeld Zoo	hairs	-	AY519452	EU004459
*Trachypithecus vetulus*	Bristol Zoo	blood, feces	-	AY519454	EU004461
*T. johnii*	Erfurt Zoo	hairs, feces	-	AY519453	EU004460
*T. pileatus*	ZMB	tissue	-	EU004472	EU004462
*T. geei*	GenBank	sequence	-	AF294618	-
*T. obscurus*	Wuppertal Zoo	blood, feces	EU004477	part of 5 kb	EU004463
*T. phayrei*	ZMB	tissue	-	AY519460	EU004464
*T. barbei*	GenBank	sequence	-	AY519462	-
*T. auratus*	Wilhelma Stuttgart	blood	-	AY519455	EU004468
*T. cristatus*	Singapore Zoo	blood, feces	-	EF465128	EU004470
*T. germaini*	ACCB	feces	-	AY519457	EU004469
*T. margarita*	GenBank	sequence	-	EF465147	-
*T. francoisi*	Bristol Zoo	hairs, feces	-	AY519458	EU004467
*T. poliocephalus*	EPRC	feces	-	EU004473	-
*T. delacouri*	EPRC	blood, feces	-	EU004474	EU004465
*T. laotum*	EPRC	blood, feces	-	EU004475	EU004466

Abbreviations: ACCB – Angkor Center for Conservation of Biodiversity, Cambodia; EPRC – Endangered Primate Rescue Center, Vietnam; MPI Leipzig – Max-Planck-Institute for Evolutionary Anthropology, Leipzig, Germany; ZMB – Zoologisches Museum Berlin, Germany

### Mitochondrial sequence analysis

To determine the phylogenetic position of langur genera among Asian colobines, a ~5 kb fragment of the mitochondrial genome was sequenced from all colobine genera with the exception of *Simias *and *Procolobus*. This region spans the cytochrome b gene, the control region, the 12S rDNA and 16S rDNA, and the intermediate tRNAs. To exclude contaminations of the dataset with nuclear pseudogenes, mainly DNA extracted from feces was used as template material, and ~1–2 kb long and ~200–400 bp overlapping fragments were amplified. PCR products were generated via hot-start technique using a set of 24 primers (Table 3) and PCR conditions comprising a pre-denaturation step at 94°C for 2 min, followed by 40 cycles at 94°C for 1 min, 60°C for 1 min and 72°C for 1–2 min, and a final extension step at 72°C for 5 min. The results of the PCR amplifications were checked on agarose gels. PCR products were cleaned with the Qiagen PCR Purification Kit and subsequently sequenced on an ABI 3100-Avant sequencer using the BigDye Terminator Cycle Sequencing Kit (Applied Biosystems). To obtain a comprehensive overview on the phylogeny of colobines, the dataset was expanded with further sequences from related taxa deposited at GenBank (Table 2). Accordingly, the final dataset comprised 15 taxa, including eight colobines, four cercopithecines, two hominoides and a New World monkey, which was used as outgroup taxon. Sequences were aligned with ClustalW [44] and subsequently checked by eye. Gaps and poorly aligned positions were removed with the G-blocks software [45], which reduced the final dataset to 4336 bp. Based on this alignment, phylogenetic trees were constructed with the maximum-parsimony (MP), neighbor-joining (NJ) and maximum-likelihood (ML) algorithms as implemented in PAUP 4.0b10 [46] and TREEPUZZLE 5.0 [47]. For MP analyses, all characters were treated as unordered and equally weighted throughout. A heuristic search was performed with the tree-bisection-reconnection (TBR) algorithm with random addition of sequences. The maximum number of trees was set to 100. NJ and ML trees were constructed with the GTR + I (= 0.2526) + Γ (= 0.4816) model of sequence evolution as it was selected as best-fitting model under the Akaike information criterion with MODELTEST 3.06 [48]. Relative support of internal nodes was performed by bootstrap analyses with 1,000 replications (MP, NJ), or by the quartet puzzling support values on the basis of 1,000 puzzling steps (ML). Finally, to evaluate the reliability of the depicted phylogenetic position of the langur genera among Asian colobines, alternative tree topologies were evaluated with the Kishino-Hasegawa [49] and Shimodaira-Hasegawa [50] tests in PAUP, both performed with full optimization and 1,000 bootstrap replications. Therefore, all three langur genera were regarded as monophyletic or variously recognized as sister clade to each other, to the odd-nosed monkey clade or even as sister clade to all other Asian colobines.

**Table 3:** Primers for amplifying and sequencing the 5 kb mitochondrial fragment.

Primer*	Sequence (5'-3')
L14182	AAACCATCGTTGTATTTCAACTA
L14621	GAGGACAAATATCATTTTGAGG
H14886	GTAGGGGTGGAAGGGAATTT
L15139	CACAAATCCAAACAACAAAGCA
H15246	ACCGGTTGGCTTCCAATTCA
L15322	CTCCTCAAATGAACTTGCCC
H15556	GCAGTAATGCACGAATTACATA
L15873	CCATCCTCCGTGAAATCAATA
H16117	TGCAGACCAGAGATAAAAGATA
L16248	GGTGTTATTTAATCCATGCTTG
H16402	TGTTTTTGGGGTTTGGCAAAG
L104	TTAGCAAGATTACACATGCAAG
H284	CATAGCTTAGTTAAACTTTCGTT
L501	CACTATGCTTAGCCCTAAACT
H572	AAGCTGTTGCTTGTAGTGTTC
L827	AAGAGTCCAAGGAGGATTTAG
H999	CCAGTACACTTACCATGTTAC
L1331	ACGAGCTACCCAAAAACAGC
H1535	TAAAGAGCTGTCCCTCTTTAG
L1608	TTAAGAAAGCGTTCAGCTCAA
H1883	TCCTTTTACTTTTTTTAACCTTTC
L2090	CCTGACCGTGCAAAGGTAG
H2342	TCCGAGGTCACCCCAACC
H2670	ATTACCGGGCTCTGCCATC

*Numbers refer to positions in the *Macaca sylvanus *mitochondrial genome, and H and L refer to the heavy and light strands, respectively.

To determine phylogenetic affiliations of species groups, a 573 bp long fragment of the mitochondrial cytochrome b gene was analysed from all species of the different groups (Table 2). The generation of sequences followed laboratory methods as described [9,11,13]. The final alignment, which was easily generated by eye due to the absence of insertions or deletions, comprised 19 individuals including the outgroup taxon *Presbytis melalophos*. Phylogenetic trees were constructed as described above. As best-fitting model, MODELTEST selected the TIM + I (= 0.5977) + Γ (= 2.3137) model, which was applied for NJ and ML reconstructions. As for the 5 kb fragment, several alternative tree topologies, in which *T*. [*vetulus*] is recognized as monophyletic, either *T*. [*vetulus*] or *T*. [*pileatus*] belongs to *Trachypithecus*, or even both are members of *Trachypithecus*, were tested.

### Y chromosomal sequence analysis

The SRY gene was selected as it represents a single-copy gene and as it is proved to be informative in reconstructing the Y chromosomal evolutionary history of macaques [51]. PCR conditions and primers were applied as described [51]. To amplify the SRY gene from fecal material, two overlapping fragments were generated with published primers [51] and the newly generated internal primers 5'-TGGGCGGAGTTGAGAGGGGT-3' and 5'-TAGCGGTCCCGTTGCTGCGG-3'. The final alignment of 777 bp comprised 15 taxa representing all species groups of the genera *Semnopithecus *and *Trachypithecus *as well as *Presbytis melalophos*, which was used as outgroup. To reconstruct NJ and ML trees, the K80 model of sequence evolution, determined with MODELTEST, was used. MP trees were generated as described above. The reliability of the depicted position of *T*. [*vetulus*] and *T*. [*pileatus*] was tested in PAUP by using alternative tree topologies, in which either *T*. [*vetulus*] belongs to *Trachypithecus *or *T. pileatus *groups with *Semnopithecus*.

### Retroposon analysis

Due to their high copy number and relative small size (~300 bp), the primate specific Alu elements were selected as cladistic markers. The presence or absence of Alu elements in different colobines at specific loci was tested via PCR using primers occupying the flanking region of the insertion site. Details on analysed loci, primers and studied species are listed in Table 4.

**Table 4:** Presence/absence analysis of retroposon insertions.

Locus	Primer (5'-3')	Presence	Absence
Asia1	AAGAATCCCAGGGAAGAACACT TTGCTGGCAAAGTGACTCCT	SENT, TOBS, PMEL, PNEM, NLAR	CGUE
Asia2	CCTGCCACTTCTGTCCATCT AGAACAAACACCAAGACAACAGC	SENT, TOBS, PMEL, PNEM, NLAR	CGUE
Asia3	GCTTTGCCACATAAAGAGCTG GGTTAGGTGCAAATGGGAAAC	SENT, TOBS, PMEL, PNEM, NLAR	CGUE
Asia4	TCAATCTTCCAGGGAAAATAAAG GAATATTAGTTGAAATATTTAGGC	SENT, TOBS, PMEL, PNEM, NLAR	CGUE
Asia5	GACCATGGTAAGACAAATGTG GACTCAGGCTTAATTTTAAGTC	SENT, TOBS, PMEL, PNEM, NLAR	CGUE
Asia6	CACCAAGCACAACTGTGAGG, TCTGCCATAGCCATCAGTCA	TOBS, PMEL, PNEM, NLAR	CGUE
Asia7	CTCTTGGTTGGGGTGAAGC GATGGTTGAACAGTGAGACTTGA	SENT, TOBS, PMEL, PNEM, NLAR	CGUE
ST1	TGATTAAAGTCAGATGAACACC GTGTAATGGGATGAAGAACAC	SENT, TVET, TDEL, TOBS, TAUR	PMEL, PNEM, NLAR
ST2	ATACATAGCATTGACTTAACTCT GATCCTGAGCCCACTATTCT	SENT, TOBS	PMEL, PNEM, NLAR
ST3	ACATCAGTGACATCAAATAAGG GAGGAAAAGATACTTTCTCATG	SENT, TOBS	PMEL, PNEM, NLAR
ST4	GGATTGAGAGCAATTTTAAAAGGA GTTCACTCCCAAATCATACTTC	SENT, TOBS	PMEL, PNEM, NLAR, CGUE
ST5	TGTAGCCAGGGAAGCCTCT TGGGATTTCTAATACTATGCCTTTG	SENT, TOBS	PMEL, PNEM, NLAR, CGUE
odd1	AGAAAGTCCCTCCCCAACAC AAGTTGGCAAAGTGGATTGC	PNEM, NLAR, RAVU	SENT, TOBS, PMEL, CGUE
T1	GAAGATTAATACTAGAAGAATCC TTGAACTTTGATCCATGGTGC	TDEL, TOBS, TAUR	TPIL, SENT, TVET, PMEL, PNEM
S1	CAAATTGTGGCTCCTTCAGTTA GGCAATGTACAGCTAACTCTGCT	SENT, TVET, TJOH	TPIL, TDEL, TOBS, TAUR, PMEL, NLAR
S2	CCCATGTGCCTTGGTTTAG GGAAGAAAGTTTGGAATGTGTG	SENT, TVET, TJOH	TPIL, TDEL, TOBS, TAUR, PMEL, NLAR

Abbreviations: CGUE – *Colobus guereza*, NLAR – *Nasalis larvatus*, PMEL – *Presbytis melalophos*, PNEM – *Pygathrix nemaeus*, RAVU – *Rhinopithecus avunculus*, SENT – *Semnopithecus entellus*, TAUR – *Trachypithecus auratus*, TDEL – *T. delacouri*, TJOH – *T. johnii*, TOBS – *T. obscurus*, TPIL – *T. pileatus*, TVET – *T. vetulus*.

To detect new loci, a subtractive hybridization approach [52] was performed with some modifications. As tracer and driver, different colobine genera were selected. Genomic DNA of tracer and driver was digested with RSAI (Fermentas), and subsequently, the adapters AdapA1/AdapAA1 (5'-TGTAGCGTGAAGACGACAGAAAGGGCGTGGTGCGGAGGGCGGT-3'/5'-ACCGCCCTCCG-3') and AdapA2/AdapAA2 (5'-TGTAGCGTGAAGACCTGTCTTAGGGCGTGGTGGCCAGGGCCGT-3'/5'-ACGGCCCTGGC-3') were ligated to the tracer fragments. Each of ~15 ng tracerA1 and tracerA2 were hybridized with ~1,500 ng driver for 20 h at 60°C. 2 μl of the hybridization result was used as template to amplify solely tracer fragments using the adapter primers A1 (5'-TGTAGCGTGAAGACGACAGAA-3') and A2 (5'-TGTAGCGTGAAGACCTGTCTT-3'). The PCR program consisted of a pre-extension step at 72°C for 6 min to fill in adaptor ends, followed by 25 cycles, each with a denaturation step at 95°C for 1 min, annealing at 60°C for 1 min and extension at 72°C for 2 min. To enrich fragments with Alu insertions, a semi-nested PCR was added using either primer A1 or A2 and the Alu-specific AluY (5'-GGAGAATGGCGTGAACCCGGGA-3') oligonueclotide. The PCR products were separated on agarose gels and fragments over 500 bp were excised from the gel. After purification, the fragments were cloned into the pGEMTeasy vector (Promega) and transformed into electro-competent TOP10 cells (Invitrogen). Bacterial clones were collected in 96-well microtiter plates and re-screened via PCR with the primers A1 or A2 and AluY. Positive clones were sequenced and analysed with REPEATMASKER and BLAST as implemented in NCBI and EMBL. Based on the generated alignments, locus-specific primers were constructed, with the forward primer occupying a region 5'-end upstream of the insertion site, which is conserved among the colobine and human or chimp sequences. Due to the absence of the 3'-end downstream sequence of the tested colobine species, reverse primers were constructed solely on the basis of human or chimp sequences. Subsequently, the presence or absence of respective Alu insertions in different colobine species was tested via PCR. The orthology of insertions was confirmed by sequencing of at least one species per genus or species group. Sequences were deposited at GenBank and are available under the accession numbers EU004484–EU004537 and EU006662–EU006692.

## Abbreviations

Species names in square brackets ([]) indicate species groups.

## Authors' contributions

MO designed primers, performed parts of the laboratory work and wrote parts of the manuscript. LW wrote parts of the manuscript and performed parts of the statistical analysis. CR collected samples, designed primers, performed parts of the laboratory and statistical work and wrote parts of the manuscript. All authors read and approved the final manuscript.
